# Supplementary material for: Cis inhibition of NOTCH1 through JAGGED1 sustains embryonic hematopoietic stem cell fate
Source: Nat Commun. 2024 Feb 21;15:1604. doi: 10.1038/s41467-024-45716-y (PMC10882055; doi:10.1038/s41467-024-45716-y)
Supplement: Supplementary file 1 — Supplementary Information [file 41467_2024_45716_MOESM1_ESM.pdf]

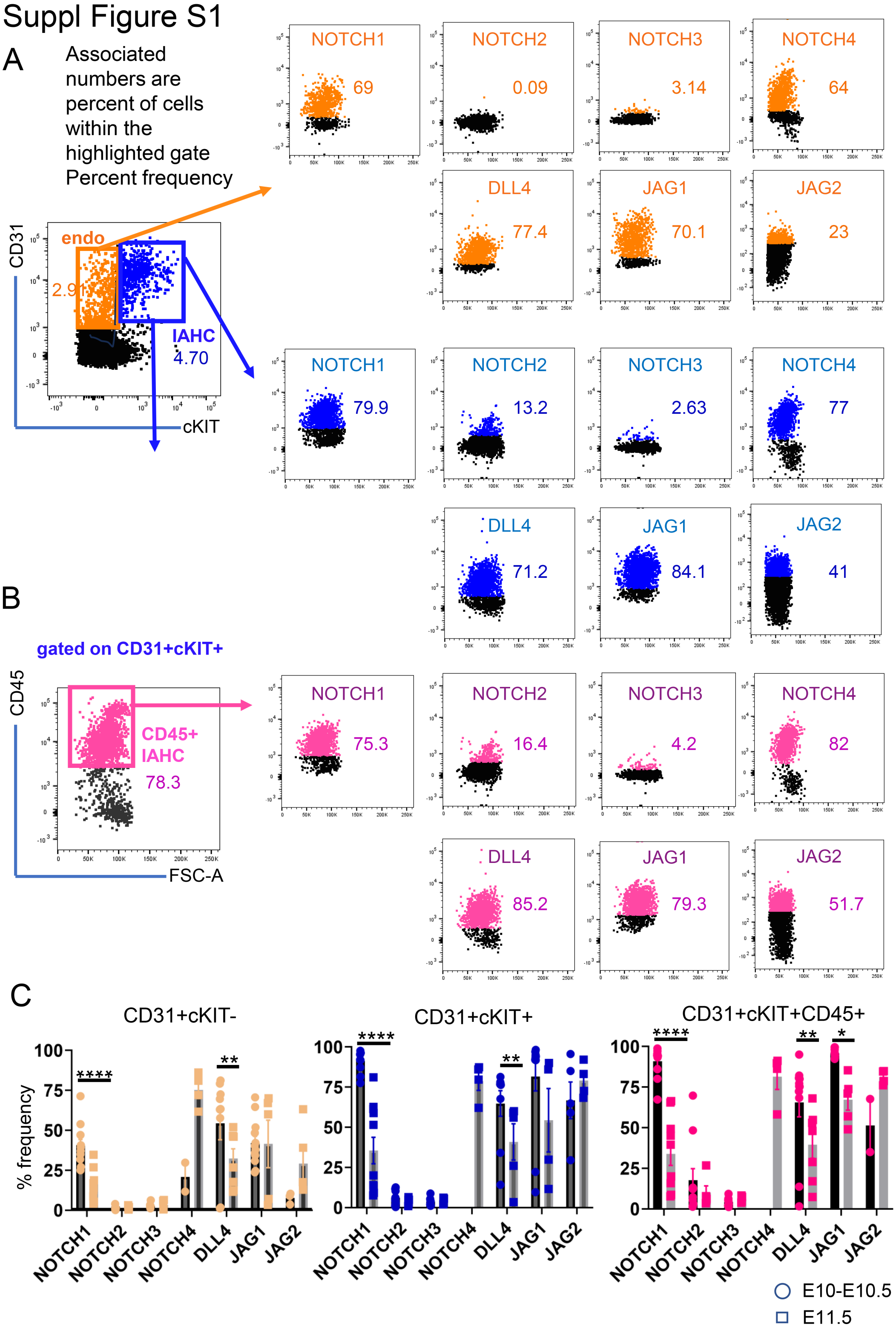

Supplementary Figure 1: Dynamic Notch signaling molecules expression patterns in the AGM (**A**) Gating for Notch receptors (NOTCH1-4) and ligands (DLL4, JAG1/2) in AGM lysates. The first gate separated endothelial cells (CD31+cKIT-, orange) from IAHC (CD31+CKIT+, blue). The positive population determined in endothelial or IAHC are highlighted in the according color. (**B**) The CD31+cKIT+ IAHC were sub-gated for CD45 expression. The notch receptor and ligand levels within the CD45 positive cells (pink) are highlighted for NOTCH1-4, DLL4 and JAG1/2. (**C**) Bar chart summarizing the expression levels for NOTCH1-4, DLL4 and JAG1/2 within CD31+cKIT- endothelium, CD31+cKIT+ IAHC and CD31+cKIT+CD45+ HSC containing IAHC (3 independent experiments with n= 41 embryos). Statistical significance was calculated with two-tailed t-tests. Vertical error bars indicate the mean and standard deviation values. Source data are provided as a source data file.

Suppl Figure S2

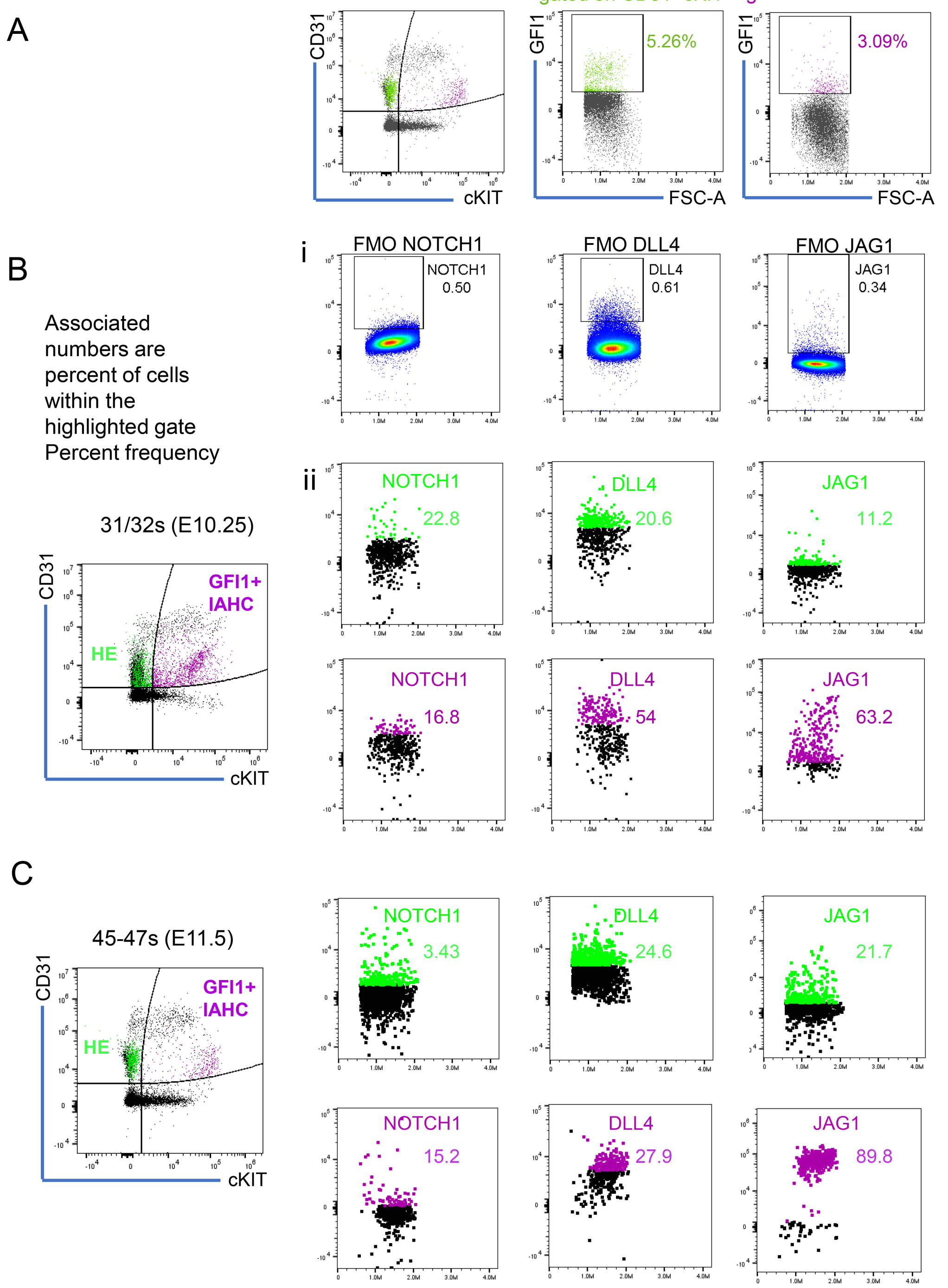

Supplementary Figure 2: GFI1 transgene expression restricts AGM population to HE and HSC containing IAHC (**A**) Gating strategy for *GFI1:tomato*+ HE (from CD31+cKIT-) and IAHC (CD31+cKIT+). The GFI1+ cells are superimposed onto the CD31/cKIT plot. (**B**) Representative Flow Cytometry plots of NOTCH1, DLL4 and JAG1. (**i**) Fluorescence minus one (FMO) control gated on CD31+ cells for NOTCH1, DLL4 and JAG1. (**ii**) GFI1 positive cells within CD31cKIT- (HE, green) and CD31+cKIT+ (GFI1+IAHC, purple) were sub gated for NOTCH1, DLL4 and JAG1 in 3132s (E10.25) AGM cell lysates. The HE and IAHC cells are superimposed onto the CD31/cKIT FACS plot. (**C**) GFI1 positive cells within CD31cKIT- (HE, green) and CD31+cKIT+ (GFI1+IAHC, purple) were sub gated for NOTCH1, DLL4 and JAG1 in 45-47s (E11.5) AGM cell lysates. The HE and IAHC cells are superimposed onto the CD31/cKIT FACS plot.

Suppl Figure S3

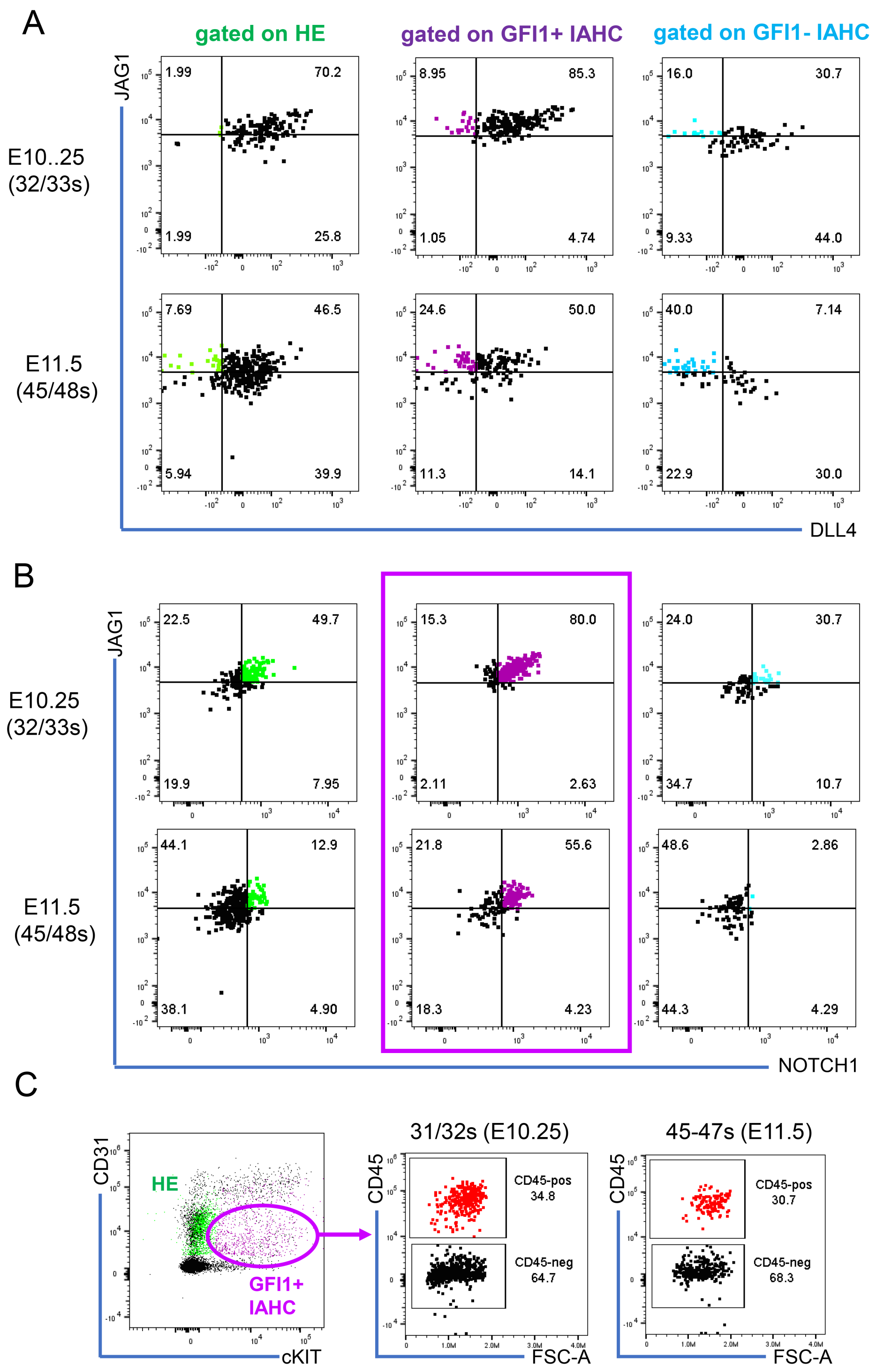

Supplementary Figure 3: GFI1+ IAHC retain NOTCH1 and JAG1 co-expression (**A**) Representative Flow Cytometry plots at 32/33s (E10.5) and 45-48s (E11.5) of CD31+cKIT-GFI1+ (HE, green), CD31+cKIT+GFI1+ (GFI1+IAHC, magenta) and CD31+cKIT+GFI1-(GFI1-IAHC, blue) for JAG1 and DLL4. (**B**) Exemplary Flow Cytometry plots at 32/33s (E10.5) and 45-48s (E11.5) of CD31+cKIT-GFI1+ (HE, green), CD31+cKIT+GFI1+ (GFI1+IAHC, magenta) and CD31+cKIT+GFI1-(GFI1-IAHC, blue) for NOTCH1 and JAG1. (**C**) Representative Flow Cytometry plots at 31/32s (E10.25) and 45-47s (E11.5) of CD31+cKIT+GFI1+ (GFI1+IAHC, magenta) for CD45.

Suppl Figure S4

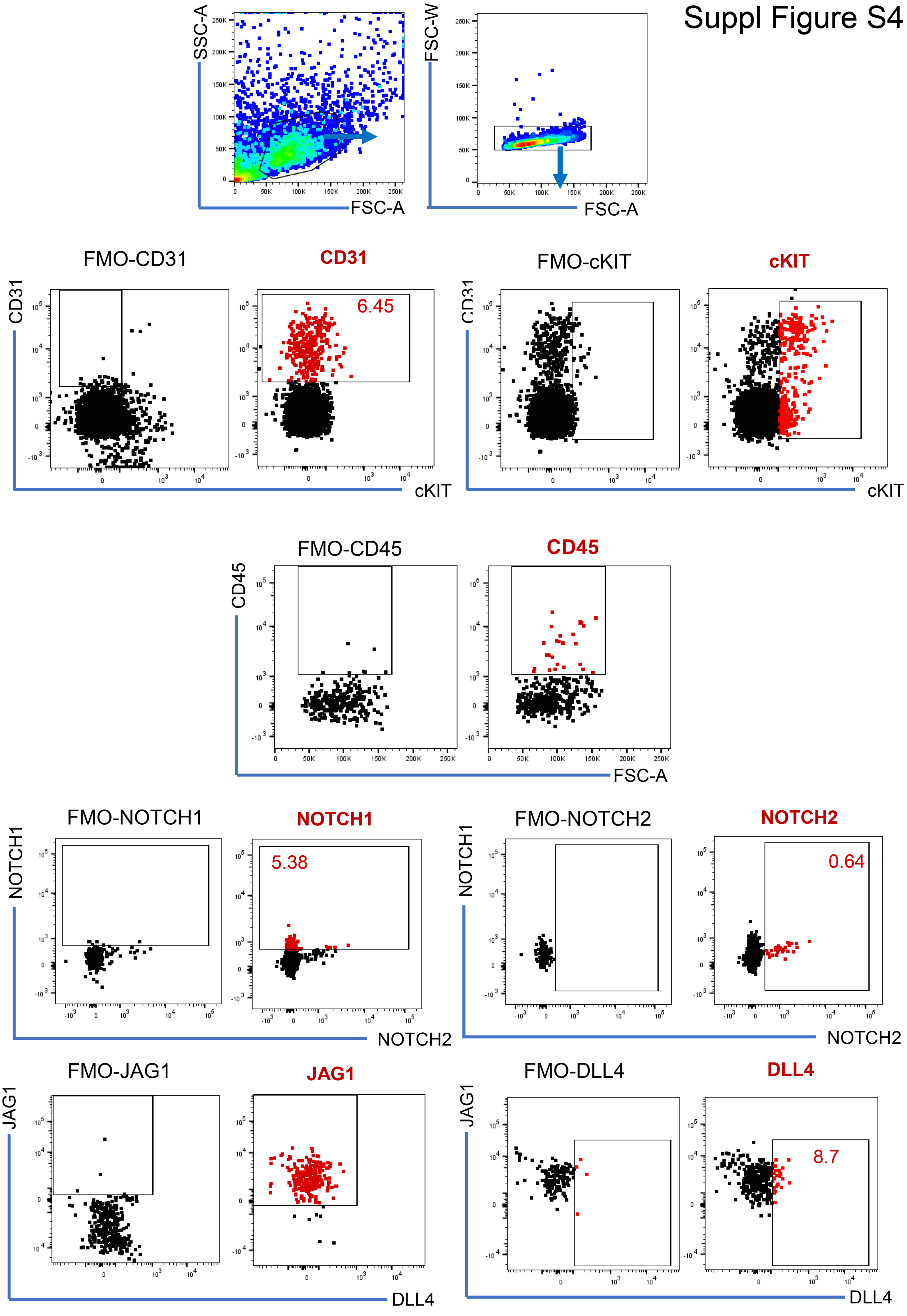

Supplementary Figure 4: Gating and Fluorescence minus one (FMO) control for FACS analysis Gating strategy to detect CD45, NOTCH1, NOTCH2, JAG1 and DLL4 in endo/HE and IAHC. FMOs were used to set up and verify gating for Flow cytometry analysis.

Suppl Figure S5

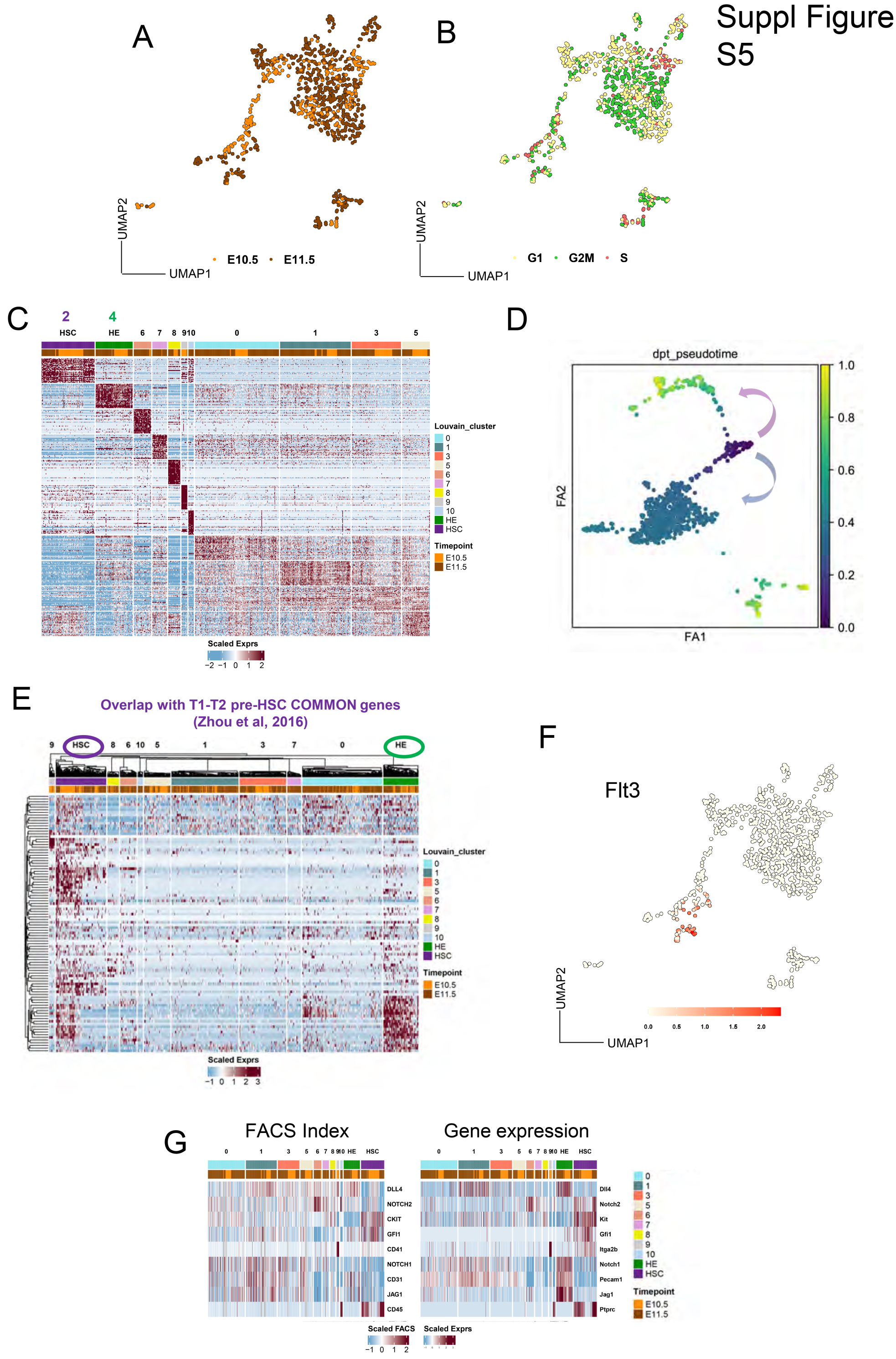

Supplementary Figure 5: The HSC cluster corresponds to the T2-HSCs (**A**) UMAP layout colored by developmental stage: E10.5 (orange, n=228 cells) and E11.5 (brown, n=547 cells). (**B**) UMAP representation of inferred cell cycle stage: G1 (yellow, n=379 cells), G2/M (green, n=313 cells) and S phase (red, n=83 cells). (**C**) Heatmap of the top 25 representative genes in each cluster. Representative genes were identified by conducting a two-sided Wilcoxon test between each cluster and the rest. Those genes not present in at least 25% cells in either groups (a particular cluster or rest) were discarded. Only those genes with adjusted p-value < 0.05 (Benjamini-Hochberg procedure for multiple testing correction) and log2 Fold Change > 0.25 were kept. The top 25 selection was based on lower adjusted p-values. (**D**) Forced directed graph layout of all sequenced cells (n=775) with colors highlighting pseudotime analysis results which was estimated considering HSC-HE cluster cells as the root. (**E**) T1/T2 common genes (98 genes) from Zhou et al, 2016 were plotted for their expression in all clusters with indication of developmental stage and identified clusters (**F**) UMAP showing the normalized expression levels of *Flt3* across (**G**) Heatmaps with Index sort levels (left) and normalized gene expression levels (left) for the indicated molecules. Developmental stage (E10.5 orange and E11.5 brown) and identified clusters annotations are included in the top banner.

Suppl Figure S6

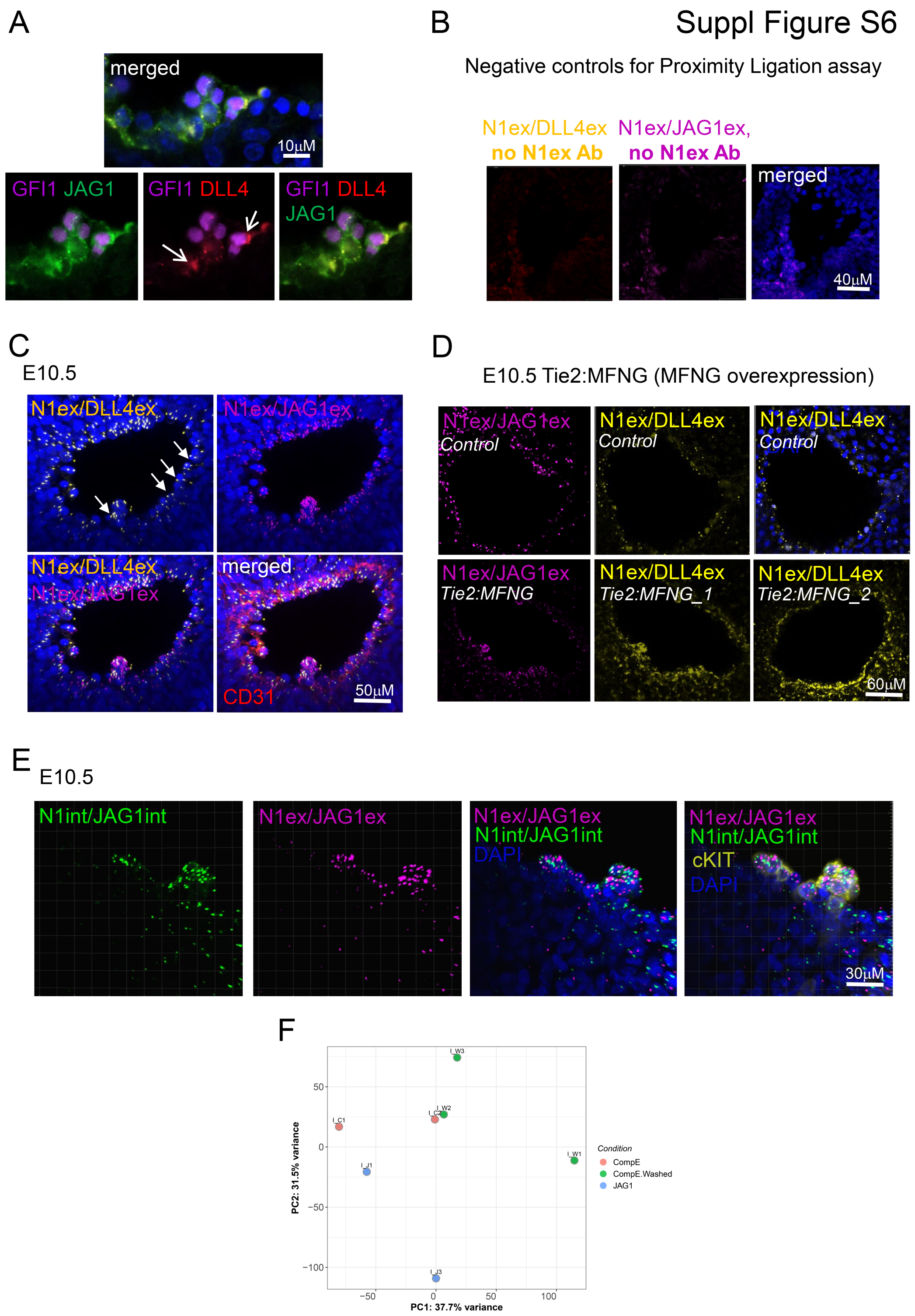

Supplementary Figure 6: cKIT positive IAHC accumulate NOTCH1-JAG1 in *cis* conformation (**A**) Immuno-histochemistry for JAG1 (green), DLL4 (red) on GFI1+Tomato (magenta) AGM sections. The localization of DLL4 at cell-cell boundaries are highlighted with a white arrow. Scale bar= 10μm. (**B**) Negative control for Proximity ligation assay. The NOTCH1ex (N1ex) antibody was omitted in this experiment. (**C**) 3D reconstruction from individual z-stacks of the dorsal aorta of E11.5 AGM with proximity ligation assay for NOTCH1ex/JAG1ex (magenta) and NOTCH1/DLL4 (yellow) with CD31 IHC (red) scale bar= 50μm. (**D**) *Tie2: Mfng* overexpressing dorsal aorta assayed with proximity ligation assay for NOTCH1ex/JAG1ex (magenta) and NOTCH1/DLL4 (yellow). Individual z-stacks of 2 different AGM aortas are shown. Scale bar= 10μm. (**E**) Compilation of z-stacks to a 3D representation of a cKIT (red) positive IAHC and DAPI (blue) probed with NOTCH1ex/JAG1ex (magenta) and NOTCH1int/JAG1int (green). Scale bar= 20um. (**F**) PC1 (37.7% data variance) and PC2 (31.5% data variance) from Principal Component Analysis (PCA) of nascent RNA data considering compE treatment (I_C1 and I_C2), after washout (I_W1-I_W3) and stimulation with Fc-JAG1 (I_J1 and I_J3) samples. Normalized expression matrix was corrected per biological sample effect prior to PCA.

Suppl Figure S7

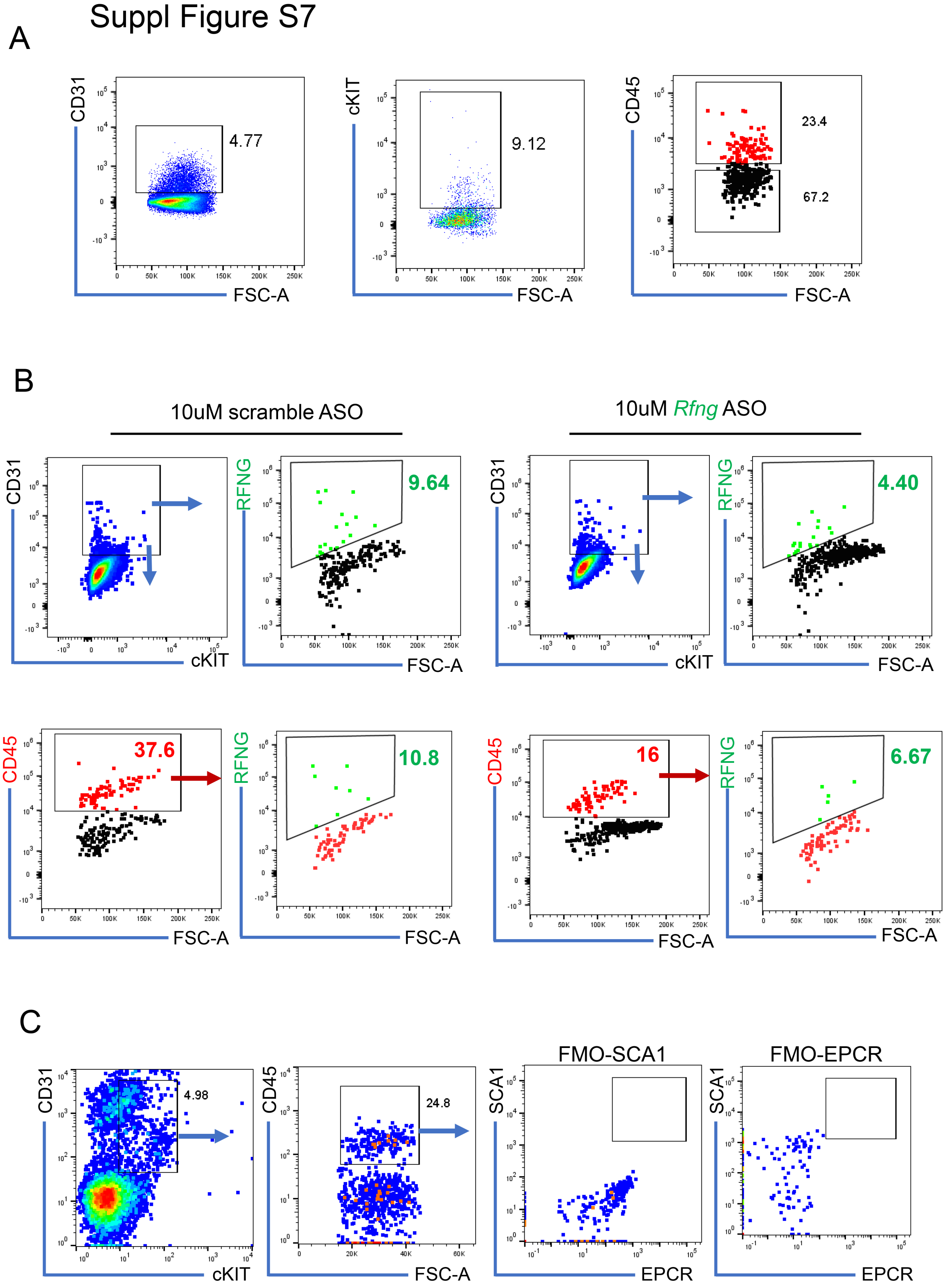

Supplementary Figure 7: Radical fringe expression modulates NOTCH1-JAG1 *cis* conformation and T2-HSC emergence (**A**) Exemplary Flow cytometry plots for determining CD45+ and CD45- IAHC. (**B**) Representative Flow cytometry plots of scramble and RFNG ASO mediated knock down in AGM explants. RFNG protein levels were assessed after gating on CD31+cKIT+ AGM cells or after further sub gating for CD45. (**C**) FMO controls for SCA1 and EPCR gates from CD31+cKIT+CD45+ cells.
