# Supplementary material for: Cis inhibition of NOTCH1 through JAGGED1 sustains embryonic hematopoietic stem cell fate
Source: Nat Commun. 2024 Feb 21;15:1604. doi: 10.1038/s41467-024-45716-y (PMC10882055; doi:10.1038/s41467-024-45716-y)
Supplement: Supplementary file 3 — Description of Additional Supplementary Files [file 41467_2024_45716_MOESM3_ESM.pdf]

Description of Additional Supplementary Files

File Name: Supplementary Data 1
Description: Antibody, primers, recombinant Jag1 and Genentech.

File Name: Supplementary Data 2
Description: This dataset gathers for each of the 11 identified clusters the following information:
a) Ranked list of genes per adjusted p-value as a result of comparing a particular cluster against the rest.
b) Pre-ranked GSEA against KEGG Pathway database to obtain enriched pathways (sorted per adjusted p-value)

File Name: Supplementary Data 3
Description: Results from an overrepresentation analysis over DEGs E11.5 against KEGG Pathways database.

File Name: Supplementary Data 4
Description: Results from an overrepresentation analysis over common DEGs (n=384 genes) from CompE and Fc-Jag1 against Washed-out comparisons against KEGG Pathway database.
