# Supplementary material for: Cis inhibition of NOTCH1 through JAGGED1 sustains embryonic hematopoietic stem cell fate
Source: Nat Commun. 2024 Feb 21;15:1604. doi: 10.1038/s41467-024-45716-y (PMC10882055; doi:10.1038/s41467-024-45716-y)
Supplement: Supplementary file 8 — Reporting Summary [file 41467_2024_45716_MOESM8_ESM.pdf]

nature portfolio

Corresponding author(s): Anna Bigas and Roshana Thambyrajah

Last updated by author(s): 2024/01/22

Reporting Summary

Nature Portfolio wishes to improve the reproducibility of the work that we publish. This form provides structure for consistency and transparency in reporting. For further information on Nature Portfolio policies, see our Editorial Policies and the Editorial Policy Checklist.

Statistics

For all statistical analyses, confirm that the following items are present in the figure legend, table legend, main text, or Methods section.

n/a	Confirmed	
☐	☒	The exact sample size (*n*) for each experimental group/condition, given as a discrete number and unit of measurement
☐	☒	A statement on whether measurements were taken from distinct samples or whether the same sample was measured repeatedly
☐	☒	The statistical test(s) used AND whether they are one- or two-sided<br>*Only common tests should be described solely by name; describe more complex techniques in the Methods section.*
☐	☒	A description of all covariates tested
☐	☒	A description of any assumptions or corrections, such as tests of normality and adjustment for multiple comparisons
☐	☒	A full description of the statistical parameters including central tendency (e.g. means) or other basic estimates (e.g. regression coefficient) AND variation (e.g. standard deviation) or associated estimates of uncertainty (e.g. confidence intervals)
☐	☒	For null hypothesis testing, the test statistic (e.g. *F*, *t*, *r*) with confidence intervals, effect sizes, degrees of freedom and *P* value noted<br>*Give P values as exact values whenever suitable.*
☒	☐	For Bayesian analysis, information on the choice of priors and Markov chain Monte Carlo settings
☒	☐	For hierarchical and complex designs, identification of the appropriate level for tests and full reporting of outcomes
☒	☐	Estimates of effect sizes (e.g. Cohen's *d*, Pearson's *r*), indicating how they were calculated

Our web collection on statistics for biologists contains articles on many of the points above.

Software and code

Policy information about availability of computer code

Data collection	scRNAseq data was collected from an Illumina NextSeq 500 sequencer platform.<br>- Nascent RNAseq data was collected from an Illumina NextSeq2000 sequencer platform<br>- FACS data was collected in a Diva (v.6.1.3) or SpectralFlow (v3.0.3) software<br>-Imaging data was collected with Leica software (v.5.1) and processed in Imarls(v.10.1)<br>- qPCRs were performed with a Lighcycler II instrument (v.1.5)
Data analysis	Regarding scRNAseq analysis:<br>- STAR aligner tool (v2.7.3)<br>- HTSeq (v0.9.1)<br>- Python3 (v3.7.3)<br>- scanpy Python toolkit (v1.4.4)<br>- anndata Python package (v0.6.22.post1)<br>- umap Python package (v0.3.10)<br>- pandas Python package (v0.25.1)<br>- scikit-learn Python module (v0.21.3)<br>- statsmodels Python package (v0.15.15)<br>- fa2 Python package (v0.3.5)<br>- R software environment (v4.2.1)<br>Jewwns 5upoda.a I oflop.Jod eJmeuryLk,1

- ggplot2 Bioconductor R package (v3.4.1)
- complexHeatmap Bioconductor R package (v2.14.0)
- Enhanced Volcano Bioconductor R package (v1.16.0)
Regarding nascent RNAseq analysis:
- FASTQC tool (v0.11.9)
- cutadapt trimming tool (v4.2)
- STAR aligner tool (v2.7.8)
- featureCounts from subRead software (v2.0.1)
- R software environment (v4.2.1)
- DESeq2 Bioconductor R package (v1.38.3)
- removeBatchEffect from limma Bioconductor R package (v3.54.2)
- clusterProfiler Bioconductor R package (v4.6.2)
- REVIGO web tool (Accessed 18th April 2023)
Other sofware used:
- GraphPad Prism 8.0.2
- FlowJO v10.7.2 CL

For manuscripts utilizing custom algorithms or software that are central to the research but not yet described in published literature, software must be made available to editors and reviewers. We strongly encourage code deposition in a community repository (e.g. GitHub). See the Nature Portfolio guidelines for submitting code & software for further information.

Data

Policy information about availability of data

All manuscripts must include a data availability statement. This statement should provide the following information, where applicable:

- Accession codes, unique identifiers, or web links for publicly available datasets
- A description of any restrictions on data availability
- For clinical datasets or third party data, please ensure that the statement adheres to our policy

Data availability
Single cell RNA-Seq and nascent RNA-seq data: GEO accession number GSE230794. Source data are provided with this paper as a Supplementary data 5.

Code availability

Custom codes used in the study are available via the GitHub repository
[https://github.com/BigaSpinosaLab/HSC_cis_inhibition_Notch1_Jag1].

Research involving human participants, their data, or biological material

Policy information about studies with human participants or human data. See also policy information about sex, gender (identity/presentation), and sexual orientation and race, ethnicity and racism.

Reporting on sex and gender	n/a
Reporting on race, ethnicity, or other socially relevant groupings	n/a
Population characteristics	n/a
Recruitment	*Describe how participants were recruited. Outline any potential self-selection bias or other biases that may be present and how these are likely to impact results.*
Ethics oversight	n/a

Note that full information on the approval of the study protocol must also be provided in the manuscript.

Field-specific reporting

Please select the one below that is the best fit for your research. If you are not sure, read the appropriate sections before making your selection.

☒ Life sciences ☐ Behavioural & social sciences ☐ Ecological, evolutionary & environmental sciences

For a reference copy of the document with all sections, see nature.com/documents/nr-reporting-summary-flat.pdf

Life sciences study design

All studies must disclose on these points even when the disclosure is negative.

Sample size	We used mouse embryonic (E10-E11.5) trunk tissues. For each experiment, at least 3 independent embryos were used.
Data exclusions	We excluded one sample (Fc-JAG1_2) from the nascent RNA sequencing data since we recovered less than 25% of unique reads for the alignment.
Replication	Each reported experiment was performed at least in two independent experiments, ie repeating the entire protocol/method. In each experiment, at least 3 individual embryos were sampled. We also used different instruments (BD Fortessa, Cytek Aurora, BD Ariall and BDInflux) and methods (FACS analysers, INC, PLA assays) to validate and cross-reference our findings.
Randomization	n/a
Blinding	n/a

Reporting for specific materials, systems and methods

We require information from authors about some types of materials, experimental systems and methods used in many studies. Here, indicate whether each material, system or method listed is relevant to your study. If you are not sure if a list item applies to your research, read the appropriate section before selecting a response.

Materials & experimental systems

n/a	Involved in the study	
☐	☒	Antibodies
☒	☐	Eukaryotic cell lines
☒	☐	Palaeontology and archaeology
☐	☒	Animals and other organisms
☒	☐	Clinical data
☒	☐	Dual use research of concern
☒	☐	Plants

Methods

n/a	Involved in the study	
☒	☐	ChIP-seq
☐	☒	Flow cytometry
☒	☐	MRI-based neuroimaging

Antibodies

Antibodies used

FACS/Index staining
name clone supplier Cat # LOT number used dilution
CD31 Alexa 647 Mec13.3 BioLegend #102515, B216399, 1/200
c-KIT—APC—eFluor780 2B8 ebioscience # 47-1171-82, 2373373, 1/200
CD45 PerCP5.5 3D-F11 Invitrogen # 45-0451-82, 2622634, 2518488, 1/200
CD2O1 (PROCR/EPCR) eFL 710 eBio1560 Invitrogen# 46-2012-80, 1187894, 1/200
Sca1-FITC E13-161.7 BD Bioscience #561077, B236533, 1/200
Notch antibodies
NOTCH1-APC HMN1-12 biolegend #130613, B284675, 1/200
NOTCH1 Alexa Fluor421 HMN1-12 biolegend #130615/200
NOTCH2-Alexa Fluor700 FAB5196-N R&D #FAB5196N-100UG, 1503007, 1/200,
NOTCH3-APC HMN3-133 ebioscience # 12-5763-80, 1942471, 1/200
NOTCH4-PE HMN4-14 ebioscience ## 12-5764-80, 4308873, 1/400
DELTA LIKE 4 -APC HMND4-1 biolegend #130813, B218775, 1/200
JAGGED1-PE HMJ1-12 biolegend #130907, B311289, 1/400
JAGGED2-bio HMJ2-1 miltenyi biotech #130-102-316, 1/200

IHC antibodies
Primary antibodies

biotinylated rat anti-mouse CD31 clone MEC13.3 BD Bioscience #553371, 1/200
rat Anti-mouse cKIT 2B8 eBioscience #13-1171-82, 2269942, 1/200
goat anti-DLL4 AF1389 R&D systems #AF1389-SP, 1/200
goat anti-JAG1 C20 santa cruz , discontinued, I2115, 1/200

Secondary antibodies
Alexa Fluor 647 goat anti-rat goat anti-rat Life Technologies #SAB4600186-250UL, 2041652, 1/400
Alexa fluor 488 donkey anti-goat donkey anti-goat Molecular probes #Cat # A-11055, 2059218, 1/400
Alexa Fluor 488 donkey anti-rabbit donkey anti-rabbit Molecular probes #Cat # A-21206, 2541645, 1/400,

Alexa Fluor 594 donkey anti-rabbit donkey anti-rabbit Life Technologies #Cat # A32740, 2266563, 1/400

Proximity Ligation assay
N1 Intracellular (Human Notch1 as 2500-2600) rabbit Anti-Notch1 antibody EP1238Y, abcam #ab52627, GR3236291, 1/400
N1 extracellular (NRR of mouse Notch1) rabbit Anti-Notch1 antibody E6 , abcam #ab245686, GR3235121, 1/400
JAG1 extracellular (aa110-125 of Human Jagged1) rabbit Anti-Jagged1 antibody recombinant, abcam #ab7771,GR288274, 1/400
JAG1 intracellular (C-terminal) goat Anti-JAG1 antibody C20 santa cruz 1/400

Antibodies kindly gifted by GENENTECH, supplied upon request
Antibody Stock concentration Working concentration
In vitro for explants
anti-Jag1, mlgG2a 8,65mg/ml 50ug/ml, LOT 27601
anti-Notch1 hIgG1 11,3mg/ml 50ug/ml, LOT 567908
Supplier catalog number concentration
Recombinant Rat Jagged 1 Fc Chimera Protein, CF R&D 44: 599-JG 4ug/ml

Validation

antibodies were validated by FACS with single staining and FMO controls. In IHC and PLA, antibodies were tested and compared in secondary only control, or by comparing the expression pattern with published data sets.

Animals and other research organisms

Policy information about studies involving animals; ARRIVE guidelines recommended for reporting animal research, and Sex and Gender in Research

Laboratory animals

Mice born in the Barrier area are socially housed, up to four male and five female mice, in 1145T (Tecniplast) cages in individually ventilated cages. Autoclaved black poplar shavings (Souralit) is used as bedding, and irradiated tissues as nesting material. Autoclaved cardboard cylinders are added to cages with mating pairs and individually-housed mice as additional environmental enrichment. Once a week mating pairs and once evry two weeks the rest of socially housed mice, together with the nesting material, are transferred to clean cages. Mice have ad libitum access to autoclaved water and irradiated diet (J. Rettenmaier & Söhne GmbH + CO KG ; RM3 for breeding pairs and young mice until nine weeks old, and RM1 as maintenance diet after nine weeks of age). Rooms are maintained under standard environmental conditions (humidity: 40–60%; temperature: 20–24℃) and a 12 h light/dark cycle (lights on at 08:00 h). Animal care and use programme is approved by PRBB-Ethics Committee and accredited by AAALAC International, following European (2010/63/UE) and Spanish (RD 53/2013) regulations.
The CD1-IGS wild type strain and Gfi1:tomato (Thambyrajah et al., 2016) were used in this study. For time matings, Gfi1:tomato or CD1 WT females were mated to Gfiltomato or CD1 WT males. Jag1floxed mice (B6.129S-Jag1tm2Grid/SjJ purchased from the Jackson laboratory) were bred to Ve-cadherin CreERT2 mice (Monvoisin et al, 2006)

Wild animals

n/a

Reporting on sex

not applicable since embryos were collected at mid-gastation stage of embryogenesis. In fact, no sex bias was introduced in this study since the gender was not determined.

Field-collected samples

n/a

Ethics oversight

n/a

Note that full information on the approval of the study protocol must also be provided in the manuscript.

Plants

Seed stocks

n/a

Novel plant genotypes

n/a

Authentication

n/a

Flow Cytometry

Plots

Confirm that:

- [x] The axis labels state the marker and fluorochrome used (e.g. CD4-FITC).
- [x] The axis scales are clearly visible. Include numbers along axes only for bottom left plot of group (a 'group' is an analysis of identical markers).
- [x] All plots are contour plots with outliers or pseudocolor plots.
- [x] A numerical value for number of cells or percentage (with statistics) is provided.

Methodology

Sample preparation	AGMs of E10- E11.5 embryos were dissected in PBS with 7% fetal calf serum (FBS) and penicillin/streptomycin (100 U/mL). Single cell suspensions were generated by incubating the tissues for 20-30 minutes in 500 ul of 1mg/ml of Collagenase/ Dispase (Roche cat# 10269638001) before mechanical dissociation with a syringe and needle. The resulting single cell suspension was used for antibody staining.
Instrument	FACS analyzers: BD Fortessa or Cytek Aurora<br>iFACS sorters: Ariall or BD Influx (BD Biosciences)
Software	FACS plots were generated using FACS Diva software or Flowio V10
Cell population abundance	FACS sorters were used in the "high purity" setting. Unfortunately, due to the small number of cells of the samples, we could not run post-sort purity test.
Gating strategy	First, using FSC-A and SSC-A we excluded cells debris. In the second step, FSC-W and FSC-A were used to gate on single cells, followed by exclusion of dead cells with DAPI staining. In the first experiment of each staining Florescence minus one controls were used to establish the cut off lines for each gate. We also cross-examined how the staining of a given positive population behaved within a known negative control population within the same sample. Finally, we also checked and compared our gates and staining efficiency with published data sets whenever possible. Frequency of a population from the previous<br>Jewwns 5upoda.a I oflop.Jod eJmeu<br>4<br>(indicated) gate are shown in each FACS plot.

- [x] Tick this box to confirm that a figure exemplifying the gating strategy is provided in the Supplementary Information.
